# Impact of cow’s milk intake on exercise performance and recovery of muscle function: a systematic review

**DOI:** 10.1186/s12970-019-0288-5

**Published:** 2019-05-06

**Authors:** Juan M. A. Alcantara, Guillermo Sanchez-Delgado, Borja Martinez-Tellez, Idoia Labayen, Jonatan R. Ruiz

**Affiliations:** 1 PROFITH “PROmoting FITness and Health through physical activity” research group Department of Physical Education and Sport, Faculty of Sport Sciences, University of Granada, Ctra. de Alfacar s/n C.P, 18071 Granada, Spain; 2 Department of Medicine, Division of Endocrinology, and Einthoven Laboratory for Experimental Vascular Medicine, Leiden University Medical Centre, Albinusdreef 2, Leiden, 2333 The Netherlands; 3 Institute for Innovation & Sustainable Development in Food Chain, Public University of Navarra, Campus Arrosadía, s/n, 31006 Pamplona, Spain

**Keywords:** Dairy product, Muscle damage, Muscle recovery, Resistance training, Endurance training

## Abstract

Dairy products are thought to improve recovery after both resistance and endurance exercises due to their nutritional proprieties. We systematically reviewed the effects of dairy product intake on exercise performance and recovery of muscle function in humans. A literature search was conducted in the MEDLINE (via PubMed) and Web of Science databases from their inception to 15th April 2018. The initial search retrieved 7708 articles, and a total of 11 studies were finally included after applying inclusion and exclusion criteria. All the selected studies were conducted with cow’s milk. Whereas some studies found significant positive effect of cow’s milk on exercise performance and recovery of muscle function, others did not find any effect. These controversies could be due to the heterogeneity of cow’s milk ingestion (e.g., amount of cow’s milk, timing of consuming the cow’s milk), to the type of intervention, and to the large heterogeneity of outcomes measured. Limited studies exist examining the effects of cow’s milk consumption and its influence on exercise performance and recovery of muscle function, therefore further studies are needed to draw more definitive conclusions.

**Electronic supplementary material:**

The online version of this article (10.1186/s12970-019-0288-5) contains supplementary material, which is available to authorized users.

## Introduction

There is evidence that proper nutritional intake is a key factor in optimizing exercise performance as well as adaptation to training (e.g., positive stimuli for protein synthesis in skeletal muscle) and recovery of muscle function (e.g. increase the recovery between training sessions or competitions, decrease the symptoms of delayed onset muscle soreness, etc.) [1, 2]. High exercise performance requires very controlled nutritional intake [3] and timing [4] before, during and after exercise to maximize exercise-induced adaptation and to shorten recovery after exercise, however the impact of either the type, composition or timing of the nutrient is still not known. Protein intake has a great impact on muscle damage repair, facilitating the recovery of muscle function (e.g. muscle strength, muscular power production, muscular stiffness, etc.) and muscular protein synthesis [1, 2]. For both hypertrophy and recovery, a positive muscle protein net balance, i.e., a higher muscle protein synthesis than muscle protein breakdown, is necessary [5].

When the rates of muscle protein synthesis and degradation increase [6, 7], an adequate nutrition is required [8–10] to facilitate the recovery process. For example, a bout of unaccustomed exercise, especially that including eccentric muscle contractions such as downhill running, can damage contractile proteins, impair muscle function, and induce muscle soreness [11, 12]. In theory, the stimulation of muscle protein synthesis by means of protein or amino acids (e.g. through dairy products ingestion) represents an important skeletal muscle adaptive response to mechanical stress that helps in recovery of muscle function [8, 13, 14].

Dairy products are rich in amino acids, proteins, lipids, minerals and vitamins, and their health benefits have been reviewed elsewhere [15]. These beneficial properties are based on the fact that dairy products, and especially cow’s milk, contains lactose (carbohydrate), casein and whey protein—commonly in a 3:1 ratio (casein:whey), as well as calcium [5, 15]. Of note is that these other nutrients present in cow’s milk such as calcium, sodium or potassium could aid in fluid recovery after exercising [5] and this improvement in the hydration state could help the recovery of the skeletal muscle. Furthermore, the aforementioned protein ratio could promote slow digestion and absorption of amino acids [5], which may lead to an increase in the serum amino acid concentration (mainly branched amino acids) [16], however, it is important to note that casein alone or whey protein alone, could increase serum amino acid concentration. These branched amino acids may have a large impact on protein synthesis and muscle metabolism [5] and therefore, helping the aforementioned muscle damage repair process. However, it is important to note that Atherton et al. [17] showed that branched amino acids effect on muscular protein synthesis is most likely due to the presence of leucine and not the presence of isoleucine or valine. Furthermore Witard et al. [18] reported that muscular protein synthesis stimulation via branched amino acids was ~ 50% inferior compared to a whey protein bolus containing similar amounts of branched amino acids. Moreover, the nutritional characteristics of dairy products (e.g. cow’s milk) [19] plus the relatively low price and high availability [20] of dairy products make them a potentially recovery-enhancing product after exercise [5]. This is observed in the current growth of scientific interest in the effects of dairy product intake on exercise performance and muscle function recovery [5].

In this systematically review, we summarize the results of the studies assessing the effect of dairy products on exercise performance and on the recovery of muscle function in humans.

## Methods

This systematic review was conducted following the Preferred Reporting Items for Systematic Reviews and Meta-Analysis (PRISMA) statement [21] and was registered through the International Prospective Register of Systematic Reviews (PROSPERO registration number: CRD42018094800).

### Search strategy

A literature search was conducted in the MEDLINE (via PubMed) and Web of Science (WOS) databases from their inception to 15th April 2018. The search terms as well as the search strategy and equations can be seen in detail in the Additional file 1: Table S1. Briefly, we used “dairy products”, “exercise”, “training”, “athletic performance”, “muscle strength”, “muscle fatigue”, and “muscle recovery” among others terms (see Additional file 1: Table S1) joined with Boolean operators. The reference lists of the retrieved systematic reviews and meta-analyses were reviewed to identify additional studies.

### Selection criteria

The inclusion criteria used were 1) dairy product and exercise intervention (either chronic or acute) studies. The difference between the intervention and the control group/period should be in the dairy product consumption. Dairy product includes raw and processed or manufactured milk and milk-derived products. Dairy products normally come from cow but could be also from goats, sheep, reindeer, and water buffalo as defined by the National Library of Medicine (PubMed) [22]; 2) conducted in healthy humans, regardless of age or fitness level; and 3) studies including measurements of exercise performance or recovery of muscle function. We included studies that measured exercise performance quantified by fitness parameters such as maximum repetition test and isokinetic dynamometry variables [23, 24]. Moreover, we included studies that assessed muscle recovery function by subjectively measurements [e.g., ratio of perceived exertion and visual analogue scales (VAS)] or objectively measured by the use of blood markers [(e.g., creatine kinase (CK) and myoglobin)] [25]. If the same data/study was used in different original articles for different purposes, only the report that provided more detailed information about the topic of this systematic review was included.

The exclusion criteria used were 1) studies written in languages other than English or Spanish; 2) studies in which any type of protein, flavoring or sweetener was added to the consumed dairy product; moreover, colostrum (e.g., bovine colostrum), chocolate milk and breast milk were excluded from this systematic review; 3) studies in which there was no a control group.

### Data extraction

The following data were collected from each included study: 1) study characteristics (author identification and reference); 2) number of participants and sex; 3) age of the participants; 4) fitness level of the participants; 5) design; 6) groups; 7) exercise intervention; 8) dairy product ingestion (e.g. cow’s milk ingestion) and placebo ingestion; 9) study outcomes; 10) results; and 11) risk of bias score.

Regarding the exercise intervention, those studies including exercises such as sprints series, isokinetic (combining eccentric and concentric contractions) or resistance (e.g. bench press) exercise or training were classified in resistance or high-intensity exercise. Those studies including exercises such as continuous cycling or cycling at different intensities (e.g. 70% peak oxygen uptake) were classified in endurance exercise.

### Study quality and risk of bias assessment

The Cochrane risk of bias tool [26] was used to evaluate the risk of bias in each study. This tool assesses random sequence generations and allocation concealment, performance bias (blinding of participants and personnel), detection bias (blinding of the outcome assessment), attrition bias (incomplete outcome data), reporting bias (selective reporting), and other sources of bias.

The literature search and data extraction as well as the quality assessment were independently performed by four reviewers (JMAA, GSD, BMT and JRR), and inconsistencies were solved by consensus.

## Results

### Overall results

**Fig. 1:**
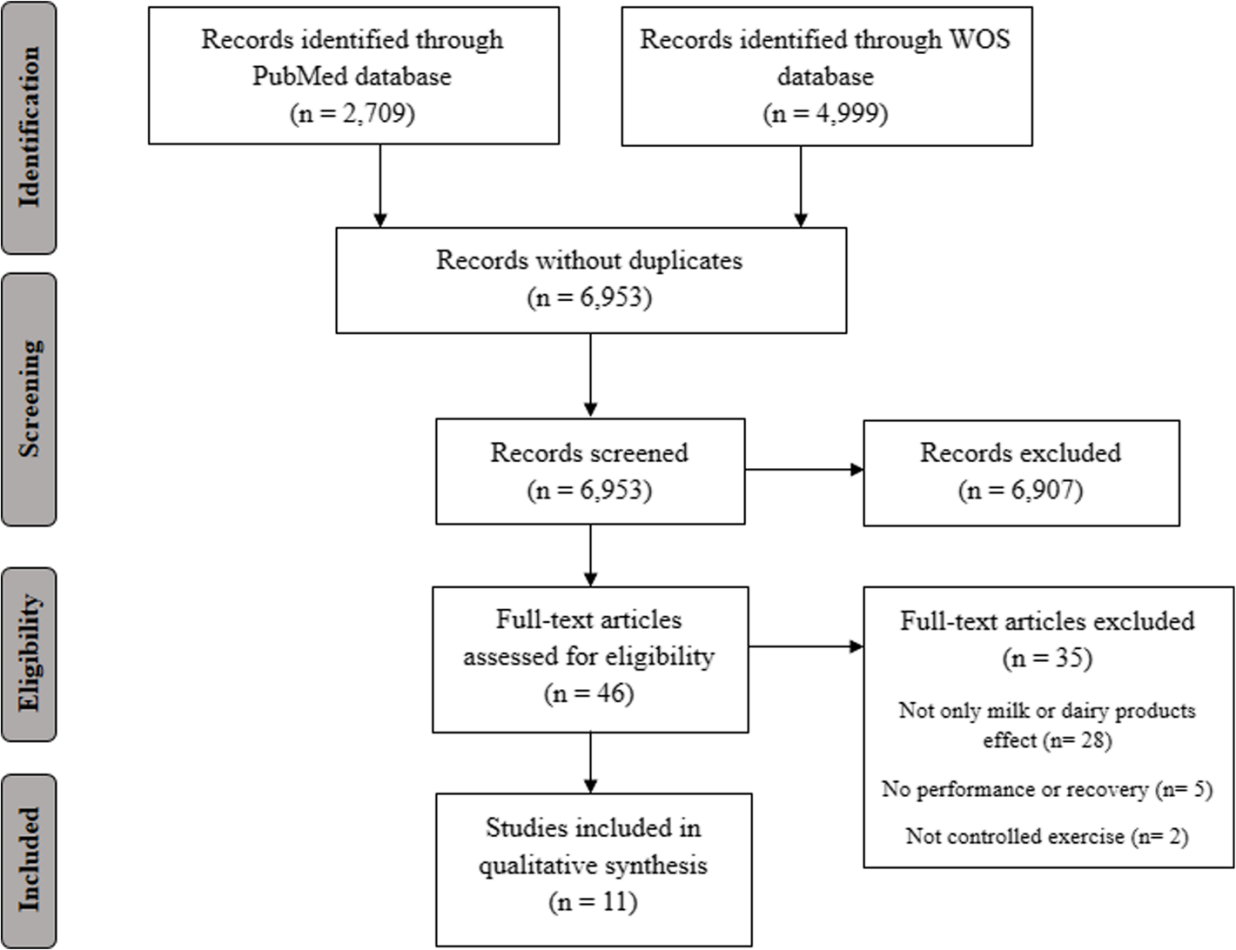
The Preferred Reporting Items for Systematic Reviews and Meta-Analyses (PRISMA) flow diagram shows the identification, screening, eligibility, and inclusion of articles in the systematic review. WOS: Web of Science

Figure 1 presents the PRISMA consort diagram for the search strategy. The initial search retrieved 7708 articles, and a total of 11 studies were finally included after applying the inclusion and exclusion criteria (Table 1). All the included studies used cow’s milk products. A total of 7 studies (63.6%) investigated the acute effect of cow’s milk after resistance or high-intensity exercise [27–33], whereas three studies (27.3%) determined the acute effect of cow’s milk after endurance exercise [34–36]. In addition, one study [37] analyzed the long-term effect (over 12 weeks of resistance training) of exercise and cow’s milk on the maximum repetition strength of squat and bench press.

**Table 1:** Overview of milk and dairy product studies on exercise performance and recovery of muscle function

Study	No. of participants (sex)	Age (years) mean ± SD	Fitness level	Design	Groups	Exercise intervention	Milk or placebo ingestion	Study outcomes	Results
Milk and acute resistance/high-intensity exercise									
Rankin et al. [28]	18 (females)	22 ± 3	Team sport athletes	Between-group design	● Cow’s low-fat milk (1%) ● Placebo energy-matched carbohydrate solution beverage (glucose and an available orange-flavored fruit cordial mixed with water)	Repeated running sprint protocol (15 × 20 m sprints) plus 8 sets of 10 plyometric jumps	500 mL immediately after the exercise. The volume of placebo was similar to the intervention one	i) peak torque of the best repetition (dominant leg); ii) RFD; iii) CMJ; iv) RSI; v) 5, 10 and 20-m sprint tests; vi) CK; vii) hsCRP; viii) passive and active VAS muscle soreness	● Cow’s milk attenuated losses in peak torque (at 60 and 180°/s for extension and flexion), CMJ, and RFD ● No effects were shown for RSI, 10 and 20-m sprints tests, VAS muscle soreness, CK and hsCRP.
Rankin et al. [27]	10 (females)	22 ± 2	Team sport athletes	Crossover design	● Cow’s low-fat milk (1%) ● Placebo energy-matched carbohydrate solution beverage (glucose and an available orange-flavored fruit cordial mixed with water)	Cycling intermittent sprint protocol (5 min warm-up, 2 x [14 × 2 min bout of exercise comprising of 10 s of passive rest, 5 s of maximal sprinting and 105 s of active recovery, with a 15 s maximal sprint followed by 1 min active recovery after the 7th and 14th 2 min bout). The exercise bouts were separated by a 10 min rest	500 mL immediately after the exercise. The volume of placebo was similar to the intervention one	i) peak torque of the best repetition (dominant leg); ii) RFD; iii) CMJ; iv) 20-m sprint test; v) CK; vi) hsCRP; vii) PC; viii) passive and active VAS muscle soreness	● Cow’s milk improved recovery of muscle function (peak torque, RFD, 20-m sprint and CMJ), inflammation and markers of muscle damage (CK, hsCRP, PC).
Rankin et al. [29]	32 (16 females, 16 males)	24 ± 4	Team sport athletes	Between-group design	● Cow’s low-fat milk (1%) ● Placebo energy-matched carbohydrate solution beverage (glucose and an available orange-flavored fruit cordial mixed with water)	Exercise inducing muscle damage in the hamstrings using isokinetic dynamometry (6 sets of 10 repetitions, eccentric and concentric contractions, with 90 s of rest between sets) at an angular speed of 60°/s	500 mL immediately after the exercise. The volume of placebo was similar to the intervention one	i) peak torque of the best repetition (dominant leg); ii) 20-m sprint; iii) CMJ; iv) CK; v) sTnI; vi) passive and active VAS muscle soreness	● Cow’s milk attenuated the decreases in peak torque and 20-m sprint and blunted the increases in passive and active VAS muscle soreness in females compared with a carbohydrate drink. ● Cow’s milk also attenuated increases in sTnI, and a similar effect on serum CK was only observed from 24 to 72 h and 48–72 h. ● In men, cow’s milk produced a minimal positive effect on soreness and muscle damage (sTnI and CK).
Cockburn et al. [32]	14 (males)	24 ± 4	Team sport athletes (semiprofessional soccer players)	Between-group design	● Cow’s emiskimmed milk (1.7%) ● Placebo beverage (water)	Exercise inducing muscle damage in the hamstrings using isokinetic dynamometry (6 sets of 10 repetitions, eccentric and concentric contractions, with 90 s of rest between sets) at a speed of 1.05 rad/s	500 mL immediately after the exercise. The volume of placebo was similar to the intervention one	i) CMJ; ii) RSI; iii) 15-m sprint test; iv) agility time; v) Loughborough Intermittent Shuttle Test; vi) CK; vii) Mb; viii) Passive and active VAS muscle soreness	● Cow’s milk improved performance on 15-m sprint test, agility time and mean 15-m sprint performance. ● There was no effect on CMJ, RSI, serum CK, serum Mb, and active and passive muscle soreness.
Cockburn et al. [30]	24 (males)	21 ± 3	Regularly competed in a variety of sports (team and individual)	Between-group design	● Cow’s semiskimmed milk (1.7%; 500 mL) ● Cow’s semiskimmed milk (1.7%; 1000 mL) ● Placebo beverage (water)	Exercise inducing muscle damage in the hamstrings using isokinetic dynamometry (6 sets of 10 repetitions, eccentric and concentric contractions, with 90 s of rest between sets) at a speed of 1.05 rad/s	500 mL or 1000 mL immediately after the exercise. The volume of placebo was 1000 mL	i) Peak torque of the best repetition (dominant leg); ii) CK; iii) Mb; iv) IL-6; v) Passive and active VAS muscle soreness	● Decrements in isokinetic muscle performance of the dominant leg and CK increases were minimized with the consumption of 500 mL of cow’s milk. ● 1000 mL of cow’s milk could blunt the increase in IL-6; however, no differences between the cow’s milk groups were observed. No other effects were observed.
Cockburn et al. [31]	24 (males)	21 ± 3	Regularly competed in team sports (football, rugby, hockey and cricket)	Between-group design	● Cow’s semiskimmed milk (1.7%) ● Low-fat chocolate milk ● Carbohydrate beverage ● Placebo beverage (water)	Exercise inducing muscle damage in the hamstrings using isokinetic dynamometry (6 sets of 10 repetitions, eccentric and concentric contractions, with 90 s of rest between sets) at a speed of 1.05 rad/s	500 mL on two occasions, immediately after and within 2 h after the exercise (total volume: 1000 mL). The volume of placebo was similar to the intervention one	i) peak torque of the best repetition (dominant leg); ii) CK; iii) Mb; iv) passive and active VAS muscle soreness	● Cow’s milk attenuated the decrements (48 h) in total work of the set, peak torque, CK and Mb after a bout of exercise-induced muscle damage. ● The muscle soreness assessed using VAS was similar between the groups.
Kirk et al. [33]	21 (males)	23 ± 3	Regularly competed in team sports (Gaelic football, soccer, rugby)	Between-group design	● A2 milk ● Regular milk (cow’s milk) ● Placebo beverage (maltodextrin mixed with water)	Repeated sprint protocol (15 × 30 m sprints with 60 s of rest between series)	500 mL immediately after the exercise. The volume of placebo was similar to the intervention one	i) CMJ; ii) MVCs; iii) 20-m sprint test; iv) VAS muscle soreness	● CMJ recovered quicker in both cow’s milk groups vs. the placebo group. No differences between groups were observed in either MVCs or VAS muscle soreness. ● There were no effects on 20-m sprint test time; however, the cow’s milk group recovered quicker than the placebo group. Moreover, relative to the baseline, decrements over 48 h were minimized in the cow’s milk vs. placebo groups.
Milk and resistance exercise intervention									
Volek et al. [37]	28 (males)	13 to 17	Not reported	Between-group design	● Cow’s fluid milk (1%) ● Placebo beverage (apple juice or grape juice depending on the week of study)	12 weeks of resistance training (1 h, 3 days/week). The program consisted of varying training loads and intensities each week, with concomitant decreasing volume	708 mL daily (plus their habitual diet). The volume of placebo was similar to the intervention one	i) RM of squat and bench press	● No differences in maximal strength (squat and bench press strength) were found between groups.
Milk and acute endurance exercise									
Upshaw et al. [34]	8 (males)	22 ± 2	Trained cyclists	Crossover design	● Cow’s low-fat milk (1%) ● Chocolate Milk (1%) ● Hemp chocolate milk ● Soy chocolate milk ● Placebo beverage (low-energy drink)	Cycling at different intensities until the participant could not continue with the appropriate cadence at an intensity of 70 and 50% of maximal power output. Afterward, a best-effort 20-km time trial test (cycloergometer)	2262 ± 148 mL (beverage plus water if applicable) immediately after the exercise and at 30 min intervals over 2 h before completing the 20-km time trial test exercise. The volume of placebo was similar to the intervention one	i) best effort 20-km time trial test; ii) HR	● Cow’s low-fat milk improved the 20-km time trial test performance vs. that of the placebo group. ● No differences in HR were observed during this test.
Lee et al. [36]	8 (males)	24 ± 4	Actives (regular physical activity)	Crossover design	● Cow’s 0.1% fat milk ● Cow’s 0.1% fat milk plus glucose ● A commercially available CHO-electrolyte sports drink ● Placebo beverage (water)	Continuous cycling exercise at an intensity of 70% VO_2peak_ until volitional exhaustion, defined as an inability to maintain a pedal cadence of ≥60 rpm	1022 ± 470 (1.5 mL/kg of body mass) every 10 min during exercise. The volume of placebo was similar to the intervention one	i) time to volitional exhaustion (exercise capacity); ii) HR; iii) expired gases; iv) RPE	● Exercise capacity, basal HR, exercise HR, expired gases and RPE were similar between groups.
Watson et al. [35]	7 (males)	23 ± 3	Actives (regular physical activity)	Crossover design	● Cow’s skimmed milk (1%) ● Placebo beverage (a commercially available carbohydrate-electrolyte drink)	Series of 10 min cycle (55 ± 6 VO_2peak_) with 5 min of resting between series, until the loss of approximately 1.8% of the initial body mass. Time to exhaustion (61 ± 4 VO_2peak_)	2263 ± 241 mL (150% of the body mass lost) during the exercise, in four equal boluses at 15 min intervals. The volume of placebo was similar to the intervention one	i) exercise to exhaustion (exercise capacity); ii) HR; iii) RPE	● No effect on time to exhaustion, VO_2_ and RPE during exercise were observed. ● HR was higher during the cow’s milk trial than during the carbohydrate trial.

*SD* Standard deviation, *RFD* Rate of force development, *CMJ* Countermovement jump, *RSI* Reactive strength index, *CK* Creatine kinase, *hsCRP* High-sensitivity C-reactive protein, *VAS* Visual analogue scales, *PC* Protein carbonyls, *sTnI* Skeletal troponin I, *Mb* Myoglobin, *IL-6* Interleukin-6, *MVCs* Maximal voluntary isometric contractions, *rad/s* radians per second, *RM* Maximum repetition, *HR* Heart rate, *RPE* Ratio of perceived exertion, *VO*_*2*_ volume of oxygen consumption, *VO*_*2peak*_ Peak oxygen uptake, *rpm* revolutions per minute

### Risk of bias within studies

**Fig. 2:**
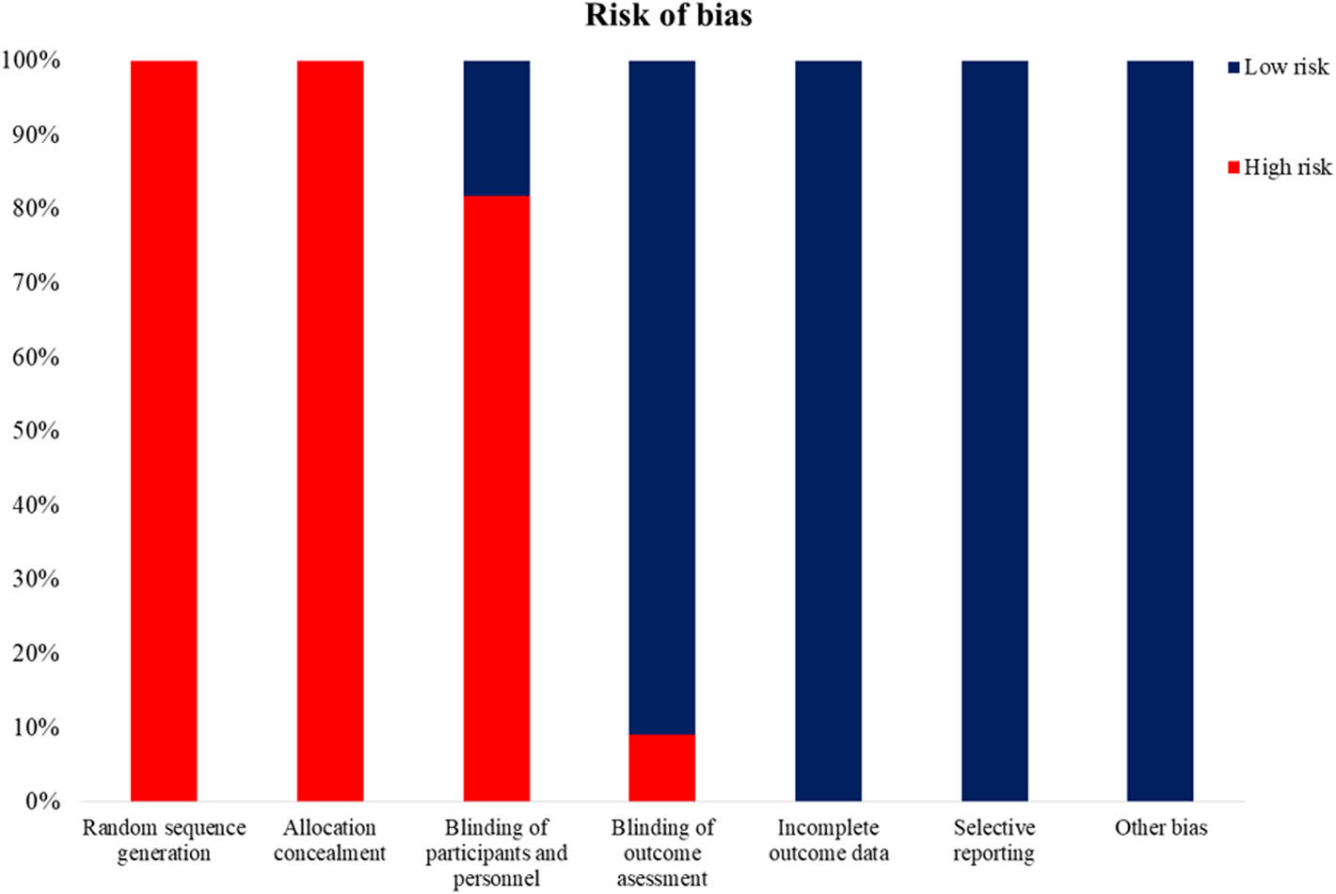
Methodological quality of the included studies. The methodological quality was assessed using the Cochrane risk of bias tool [26]

The quality of the included studies was predominantly suboptimal. The methodological quality assessment is shown in Fig. 2. Details of randomization [27–37], allocation concealment [27–37], and study blinding [27–32, 35–37] were inadequately reported or rated as an “unclear risk” (categorized when the information was not specified in the article) for most studies.

### Effects of cow’s milk on performance and muscle function recovery after resistance or high-intensity exercise

The exercise performance outcomes measured were muscular strength related variables [27–33, 37]. Regarding muscle function recovery, most of the studies [27–33] determined muscle soreness or damage using subjective scales such as VAS, and the most of them added also measures of blood biomarkers (e.g., CK or myoglobin) [27–32].

Cow’s milk attenuated losses in peak torque (maximal effort concentric knee flexion) [27, 28, 30, 31], total work of the set (6 concentric knee flexion repetitions) using isokinetic dynamometry [31], countermovement jump [27, 28, 33], rate of force development of an isometric contraction of the dominant leg quadriceps [27, 28], and sprint tests [27, 29, 32]. On the other hand, other studies observed no effect of cow’s milk on sprint recovery [28, 33], countermovement jump [32], reactive strength index [32], and bench and squat maximum strength after an exercise intervention [37].

Cow’s milk did not modify the pre-post resistance exercise changes in serum CK [27, 28, 32], myoglobin [32], high-sensitivity C-reactive protein [27, 28] and protein carbonyls [27]. In contrast, a positive effect, i.e. lower increase in CK and myoglobin concentrations, was observed from the baseline to 48 h after resistance exercise in the cow’s milk group compared with the values of the placebo beverage group [31]. Cockburn et al. [30] also showed that the increase in CK can be blunted after resistance exercise with less cow’s milk ingestion (500 mL of cow’s milk instead of 1000 mL of cow’s milk). These lower increases of CK were observed from 24 to 72 h after exercise-induced muscle damage in the hamstring and cow’s milk ingestion [29]. Cow’s milk also attenuated the skeletal troponin I increase after exercise compared with a placebo group (energy-matched carbohydrate solution) [29].

Cow’s milk did not improve muscle soreness after resistance exercise in other studies [31–33], whereas it had a positive effect on muscle soreness and tiredness at 72 h post resistance exercise in other [27]. Moreover, cow’s milk reduced passive soreness in males and females, as well as active muscle soreness (all from baseline to 72 h) in both sexes [29]. Similar results were found in another study that compared cow’s milk vs. energy-matched carbohydrate solution as a control at 72 h [28]. Finally, passive measurements of muscle soreness (using VAS) showed a benefit of limiting increases in muscle soreness in the group receiving less bolus cow’s milk (500 mL) compared with the high-bolus cow’s milk group (1000 mL) between the baseline and 48 h after exercise and cow’s milk ingestion [30]. Due to lack of homogeneity in the measurement of exercise performance, and on the recovery of muscle function outcomes after resistance or high-intensity exercise intervention doing a meta-analysis was not possible (see Table 1).

### Effects of cow’s milk on exercise performance and muscle function recovery after endurance exercise

Cow’s milk before (2 h prior the exercise) endurance exercise improved performance in a 20-km time trial (*P* < 0.05) [34]. Moreover, no differences in heart rate were observed between the cow’s milk group and the placebo group [34]. In another study [35], the mean cycling time to exhaustion was the same on the placebo group trial (39.6 ± 7.3 min) compared with the cow’s milk group (39.7 ± 8.1 min; *P* = 0.879). Furthermore, no differences in oxygen consumption during exercise were found [35]. Finally, there was no effect of cow’s milk on changes in the rate of perceived exertion after exercise (*P* = 0.744) compared with the placebo group [35].

Lee et al. [36] did not observe differences in the time to volitional exhaustion independently of the beverage ingested (median, range: 103.3, 85.7–228.5 vs. 93.3, 82.4–192.3 min for the cow’s milk vs. placebo group, respectively) [36]. Cow’s milk did not alter the heart rate reached in the volitional exhaustion point nor the RPE sensations during the exercise [36]. Due to heterogeneity in the measurement of exercise performance and on the recovery of muscle function outcomes after endurance exercise doing a meta-analysis was not possible (see Table 1).

## Discussion

We systematically reviewed and summarized the results of the studies investigating the effects of dairy products on exercise performance or on the recovery of muscle function in humans. All the studies meeting the criteria were conducted using cow’s milk. The studies investigating the effect of cow’s milk after high-intensity exercise [27–33] or resistance training [37] reported contradictory results. Whereas some studies found significant effects of cow’s milk intake on performance or recovery of muscle function such as attenuated losses in peak torque, countermovement jump, rate of force development, sprint series and inflammation and markers of muscle damage, others did not find any effect. Therefore, there is currently not enough evidence to conclude whether cow’s milk has a positive effect on exercise performance and recovery of muscle function, and further studies are needed to make more definitive conclusions. The results regarding endurance exercise are similar to those observed in resistance exercise. Whereas one study observed significant effect of cow’s milk intake on performance [34] others [35, 36] did not observe any enhancing effect. The observed contradictory findings can be explained by the heterogeneity of cow’s milk ingestion, such as the amount of cow’s milk, the timing of the cow’s milk intake, and the type of intervention, as well as by the large heterogeneity of outcomes measured. Moreover, the study participants’ fitness level may also play an important role when studies are compared. In addition, doing a meta-analysis was not appropriate due to the heterogeneity between studies, which was mainly identified in the reporting of exercise performance and on the recovery of muscle function.

It has been shown that 20 g of protein might be sufficient to stimulate muscle protein synthesis after resistance exercise [38], so perhaps a greater consumption of protein (via cow’s milk) would have resulted in more positive effects. It is noteworthy, however, that one study showed similar effects after consumption of 500 and 1000 mL of cow’s milk [30]. Volek et al. [37] found no differences between the effects of cow’s milk vs. juice (both groups consumed 708 mL daily) after a resistance exercise intervention in maximum repetition strength (12 weeks of resistance training). Both Lee et al. [36] and Watson et al. [35] found no effect of cow’s milk on time to volitional exhaustion, heart rate, expired gases and RPE after endurance exercise. In contrast, Upshaw et al. [34] found that low-fat cow’s milk (2262 ± 299 mL) improved the time in a 20-km time trial test after glycogen-lowering exercise compared with that of a placebo group (2262 ± 290 mL low-energy drink). Regarding the intensity of the exercise, one study [35] reported that the heart rate during endurance exercise in a cow’s milk trial test (2263 ± 241 mL) was higher than that during a carbohydrate trial test (2280 ± 249 mL), yet no differences in oxygen consumption during exercise were observed. The intensity and exercise performed in both studies was similar [35, 36], while in the study by Upshaw et al. [34], a glycogen-lowering exercise was performed before both cow’s milk ingestion and the examined exercise. The amount of cow’s milk ingested in the study by Upshaw et al. [34] and in the study by Watson et al. [35] was similar, and therefore, the differences in recovery between the groups might be partially explained by differences in the fitness levels of the participants [35, 36]. In the study by Upshaw et al. [34], the participants were trained cyclists, while in the other study, the participants were regularly active individuals.

Philips et al. [39] in their review focused on the evidence showing the differences in responses of muscle protein synthesis and muscle protein accretion in humans, concluded that cow’s milk-based proteins (whey and casein) appear to be better than carbohydrate beverages in the promotion of hypertrophy. Moreover, they highlighted the importance of the dose-response in the studies since the difference in leucine content (as is present in cow’s milk) may have an important influence in maintaining and possibly increasing the muscle mass [39]. Furthermore, leucine could have an impact in the recovery process (e.g. in the muscle protein synthesis and muscle protein accretion). Maybe the contradictory results obtained in exercise performance in our systematic review could be partially explained by the different amount of cow’s milk (and therefore leucine content) provided in the selected studies.

It is assumed that a single cow’s milk bolus intake increases net amino acid synthesis in young healthy sedentary volunteers [40]. Moreover, the consumption of protein and carbohydrates together, as is presented in cow’s milk, results in a higher rate of protein synthesis compared to that from the intake of these nutrients separately [41, 42]. Regarding protein consumption, sufficient protein intake is necessary to stimulate protein synthesis as we mentioned previously [38]. Therefore, it is biologically plausible that cow’s milk consumption after a bout of exercise can stimulate protein synthesis metabolism [6, 7]. Muscle membrane damage following exercise occurs as a result of mechanical stress during the first phase of muscular damage, with further disruption via the lysosomal pathway during the next phase [43]. The lack of a positive effect of cow’s milk on some blood markers (e.g., protein carbonyls and high-sensitivity C-reactive protein) [27, 28, 32] may suggest that the ingestion of cow’s milk could affect other metabolic pathways. For example, the CK concentration increases after exercise, but no clear CK blunting effects were observed from cow’s milk, while the skeletal troponin I rise was blunted in the cow’s milk group [29]. Controversially, in another study it was shown that the CK increase can be attenuated with lower cow’s milk intake (500 mL instead of 1000 mL) [30], and an effect on myoglobin was also observed between the baseline and 48 h after exercise [31] from cow’s milk ingestion. Of note is that in both of these studies, participants played regularly in team sports, and this fact could result in less muscle damage due their fitness level. In their review, Sousa et al. [44] recommend the ingestion of 0.8–1.2 g carbohydrate/kg/h and 0.2–0.4 g protein/kg/h preferably after the exercise, with a minimum of 20 g high-quality protein for improve the recovery after exercise. However, some controversial still exist regarding the correct timing and if carbohydrate and protein have to be consumed right after the exercise [44, 45]. Following these recommendations by Sousa et al. [44], maybe the negative results found in some of the included studies could be partially explained for either an insufficient amount of cow’s milk (i.e. not enough protein and/or leucine) or the timing of ingestion. Regarding the time of ingestion, most of the studies provided the beverage (e.g. cow’s milk, placebo, etc.) immediately after the exercise [27–32], while others offered the beverage during the exercise [35, 36], and one [34] offered the beverage immediately after the exercise and every 30 min. In Volek et al. [37] the beverage consumption was daily.

Regarding muscle soreness perception, whereas several studies did not find a positive effect of cow’s milk [31–33, 36], others observed significant differences in both active and passive muscle soreness between cow’s milk and control groups (~ 500 mL) [27–30]. However, VAS muscle soreness perception is a more subjective outcome than blood markers, and it is more difficult to establish whether these differences could be explained by the treatment (e.g., cow’s milk vs. placebo), by the physical condition of participants (e.g., team sport players vs. individuals not habituated to exercise), or by unaccustomed sensations after resistance (i.e., repeated eccentrics contractions).

The current review has several limitations. First, there is a high degree of heterogeneity among the analyzed studies, in part due to differences in the type, intensity, volume, frequency, and duration of the interventions, as well as in the outcome measures, and, for these reasons a meta-analysis was not possible. This review is also limited by the suboptimal methodological quality of the included interventions. Finally, because the search was limited to articles published in English or Spanish and gray literature was not consulted, the language restrictions and unpublished studies might slightly modify our results. Therefore, the results should be taken with caution, and more research on the effects of cow’s milk and dairy products is required before definitive recommendations can be provided.

## Conclusions

In conclusion, based on the current evidence, it cannot be determined whether cow’s milk has a positive effect on exercise performance and recovery of muscle function in humans, due to the limited number of studies included in this systematic review. Nevertheless, since cow’s milk is a source of protein, carbohydrates, calcium and other nutrients, and thus may lead to an increase in the serum amino acid concentration and, therefore, helping the muscle damage repair process. In line with this, some studies included found significant effects of cow’s milk intake on performance and recovery of muscle function. For these reasons, more and better study designs such as blinding the beverage to both, participants and personnel, generate a random sequence of beverage group, etc. are needed to demonstrate its usefulness as a sport nutrition-related supplement.

## Additional file

Additional file 1: **Table S1.** Search equations for both databases. (DOCX 14 kb)

## Abbreviations

CK: Creatine kinase
CMJ: Countermovement jump
HR: Heart rate
hsCRP: High-sensitivity C-reactive protein
IL-6: Interleukin-6
Mb: Myoglobin
MeSH: Medical subject heading
MVCs: Maximum voluntary isometric contractions
PC: Protein carbonyls
Rad/s: Radians per second
RFD: Rate of force development
RM: Maximum repetition
RPE: Ratio of perceived exertion
RPM: Revolutions per minute
RSI: Reactive strength index
sTnI: Skeletal troponin I
VAS: Visual analogue scales
VO_2_: Volume of oxygen consumption
VO_2peak_: Peak oxygen uptake
WOS: Web of Science

## References

[1] Pennings B, Koopman R, Beelen M, Senden JMG, Saris WHM, van Loon LJC (2011). Exercising before protein intake allows for greater use of dietary protein-derived amino acids for de novo muscle protein synthesis in both young and elderly men. Am J Clin Nutr.

[2] Yang Y, Breen L, Burd NA, Hector AJ, Churchward-Venne TA, Josse AR (2012). Resistance exercise enhances myofibrillar protein synthesis with graded intakes of whey protein in older men. Br J Nutr.

[3] Ziegenfuss TN, Landis JA, Lemieux RA (2010). Protein for sports-new data and new recommendations. Strength Cond J.

[4] Areta JL, Burke LM, Ross ML, Camera DM, West DWD, Broad EM (2013). Timing and distribution of protein ingestion during prolonged recovery from resistance exercise alters myofibrillar protein synthesis. J Physiol.

[5] Roy BD (2008). Milk: the new sports drink? A review. J Int Soc Sports Nutr.

[6] Pitkanen HT, Nykanen T, Knuutinen J, Lahti K, Keinanen O, Alen M, Komi PV, Mero AA (2003). Free amino acid Pool and muscle protein balance after resistance exercise. Med Sci Sports Exerc.

[7] Biolo G, Maggi SP, Williams BD, Tipton KD, Wolfe RR (1995). Increased rates of muscle protein turnover and amino acid transport after resistance exercise in humans. Am J Physiol Metab.

[8] Levenhagen DK, Carr C, Carlson MG, Maron DJ, Borel MJ, Flakoll PJ (2002). Postexercise protein intake enhances whole-body and leg protein accretion in humans. Med Sci Sports Exerc.

[9] Koopman R, Wagenmakers AJM, Manders RJF, Zorenc AHG, Senden JMG, Gorselink M (2005). Combined ingestion of protein and free leucine with carbohydrate increases postexercise muscle protein synthesis in vivo in male subjects. Am J Physiol Metab.

[10] Rasmussen BB, Tipton KD, Miller SL, Wolf SE, Wolfe RR (2000). An oral essential amino acid-carbohydrate supplement enhances muscle protein anabolism after resistance exercise. J Appl Physiol.

[11] Clarkson PM, Nosaka K, Braun B (1992). Muscle function after exercise-induced muscle damage and rapid adaptation. Med Sci Sports Exerc.

[12] Pasiakos SM, Lieberman HR, McLellan TM (2014). Effects of protein supplements on muscle damage, soreness and recovery of muscle function and physical performance: a systematic review. Sports Med.

[13] Moore DR, Stellingwerff T (2012). Protein ingestion after endurance exercise: the “evolving” needs of the mitochondria?. J Physiol.

[14] Howarth KR, Moreau NA, Phillips SM, Gibala MJ (2009). Coingestion of protein with carbohydrate during recovery from endurance exercise stimulates skeletal muscle protein synthesis in humans. J Appl Physiol.

[15] Haug A, Høstmark AT, Harstad OM (2007). Bovine milk in human nutrition – a review. Lipids Health Dis.

[16] Bos C, Metges CC, Gaudichon C, Petzke KJ, Pueyo ME, Morens C (2003). Postprandial kinetics of dietary amino acids are the Main determinant of their metabolism after soy or Milk protein ingestion in humans. J Nutr.

[17] Atherton PJ, Smith K, Etheridge T, Rankin D, Rennie MJ (2010). Distinct anabolic signalling responses to amino acids in C2C12 skeletal muscle cells. Amino Acids.

[18] Witard OC, Jackman SR, Breen L, Smith K, Selby A, Tipton KD (2014). Myofibrillar muscle protein synthesis rates subsequent to a meal in response to increasing doses of whey protein at rest and after resistance exercise. Am J Clin Nutr.

[19] Fogelholm M (2003). Dairy products, meat and sports performance. Sports Med.

[20] Hoffman JR, Falvo MJ (2004). Protein - Which is Best?. J Sports Sci Med.

[21] Moher D, Liberati A, Tetzlaff J, Altman DG, PRISMA Group (2009). Preferred reporting items for systematic reviews and meta-analyses: the PRISMA statement. BMJ.

[22] National Library of Medicine. Medical Subject Headings. MeSH Descriptor Data. 2018 [cited 2018 June 12]. Available from: https://www.ncbi.nlm.nih.gov/mesh/?term=dairy+product

[23] McMaster DT, Gill N, Cronin J, McGuigan M (2014). A brief review of strength and ballistic assessment methodologies in sport. Sports Med.

[24] Paul DJ, Nassis GP (2015). Testing strength and power in soccer players. J Strength Cond Res.

[25] Brancaccio P, Lippi G, Maffulli N (2010). Biochemical markers of muscular damage. Clin Chem Lab Med.

[26] Higgins JPT, Green S. Cochrane handbook for systematic reviews of interventions version 5.1.0 [updated March 2011]: The Cochrane Collaboration; 2011. Available from www.cochrane-handbook.org.

[27] Rankin P, Lawlor MJ, Hills FA, Bell PG, Stevenson EJ, Cockburn E (2018). The effect of milk on recovery from repeat-sprint cycling in female team-sport athletes. Appl Physiol Nutr Metab.

[28] Rankin P, Landy A, Stevenson E, Cockburn E (2018). Milk: an effective recovery drink for female athletes. Nutrients.

[29] Rankin P, Stevenson E, Cockburn E (2015). The effect of milk on the attenuation of exercise-induced muscle damage in males and females. Eur J Appl Physiol.

[30] Cockburn E, Robson-Ansley P, Hayes PR, Stevenson E (2012). Effect of volume of milk consumed on the attenuation of exercise-induced muscle damage. Eur J Appl Physiol.

[31] Cockburn E, Hayes PR, French DN, Stevenson E, St Clair Gibson A (2008). Acute milk-based protein–CHO supplementation attenuates exercise-induced muscle damage. Appl Physiol Nutr Metab.

[32] Cockburn E, Bell PG, Stevenson E (2013). Effect of Milk on team sport performance after exercise-induced muscle damage. Med Sci Sports Exerc.

[33] Kirk B, Mitchell J, Jackson M, Amirabdollahian F, Alizadehkhaiyat O, Clifford T (2017). A2 Milk enhances dynamic muscle function following repeated Sprint exercise, a possible ergogenic aid for A1-protein intolerant athletes?. Nutrients.

[34] Upshaw AU, Wong TS, Bandegan A, Lemon PW (2016). Cycling time trial performance 4 hours after glycogen-lowering exercise is similarly enhanced by recovery nondairy chocolate beverages versus chocolate Milk. Int J Sport Nutr Exerc Metab.

[35] Watson P, Love TD, Maughan RJ, Shirreffs SM (2008). A comparison of the effects of milk and a carbohydrate-electrolyte drink on the restoration of fluid balance and exercise capacity in a hot, humid environment. Eur J Appl Physiol.

[36] Lee JKW, Maughan RJ, Shirreffs SM, Watson P (2008). Effects of milk ingestion on prolonged exercise capacity in young, healthy men. Nutrition.

[37] Volek JS, Gómez AL, Scheett TP, Sharman MJ, French DN, Rubin MR (2003). Increasing fluid milk favorably affects bone mineral density responses to resistance training in adolescent boys. J Am Diet Assoc.

[38] Moore DR, Robinson MJ, Fry JL, Tang JE, Glover EI, Wilkinson SB (2009). Ingested protein dose response of muscle and albumin protein synthesis after resistance exercise in young men. Am J Clin Nutr.

[39] Phillips SM, Tang JE, Moore DR (2009). The role of milk- and soy-based protein in support of muscle protein synthesis and muscle protein accretion in young and elderly persons. J Am Coll Nutr.

[40] Elliot TA, Cree MG, Sanford AP, Wolfe RR, Tipton KD (2006). Milk ingestion stimulates net muscle protein synthesis following resistance exercise. Med Sci Sports Exerc.

[41] Børsheim E, Cree MG, Tipton KD, Elliott TA, Aarsland A, Wolfe RR (2004). Effect of carbohydrate intake on net muscle protein synthesis during recovery from resistance exercise. J Appl Physiol.

[42] Miller SL, Tipton KD, Chinkes DL, Wolf SE, Wolfe RR (2003). Independent and combined effects of amino acids and glucose after resistance exercise. Med Sci Sports Exerc.

[43] Armstrong RB (1990). Initial events in exercise-induced muscular injury. Med Sci Sports Exerc.

[44] Sousa M, Teixeira VH, Soares J (2014). Dietary strategies to recover from exercise-induced muscle damage. Int J Food Sci Nutr.

[45] Schoenfeld BJ (2012). Does exercise-induced muscle damage play a role in skeletal muscle hypertrophy?. J Strength Cond Res.

