# Supplementary material for: Impact of cow’s milk intake on exercise performance and recovery of muscle function: a systematic review
Source: J Int Soc Sports Nutr. 2019 May 6;16:22. doi: 10.1186/s12970-019-0288-5 (PMC6503439; doi:10.1186/s12970-019-0288-5)
Supplement: Supplementary file 1 — Table S1. Search equations for both databases. (DOCX 14 kb) [file 12970_2019_288_MOESM1_ESM.docx]

**Additional file**

**Table S1.** Search equations for both databases.

| **Database** | **Search equation** |
| --- | --- |
| PubMed | ((((((("dairy products"[Mesh] OR "dairy" OR "milk" OR "yogurt" OR "cheese" OR “kefir” OR “butter”) AND (“Exercise” [Mesh] OR “Training” OR “muscles” [Mesh] OR “muscle strength” [Mesh] OR “muscle fatigue” OR “Athletic Performance” [Mesh] OR “muscle recovery” OR “muscle function”)))) NOT (((((((((((((((((("Mice"[Mesh]) OR "Rats"[Mesh]) OR "Animal Experimentation"[Mesh]) OR "Models, Animal"[Mesh])) OR ("rats" OR "mouse"))) OR "mice")) OR "rat")))))))))))) NOT breastfeeding |
| WOS | (Dairy product* OR Dairy OR Milk OR Yogurt OR Cheese OR Kefir OR Butter) AND (Exercis* OR Training OR Muscl* OR “Muscle strength” OR “Muscle Fatigue” OR “Athletic Performance” OR “Muscle Recovery” OR “Muscle Function”) NOT (Mice OR Rat* OR (Experiment* AND Animal*) OR (Research* AND Animal*) OR mouse OR (model* AND animal*)) NOT Breastfeeding |

Mesh: Medical Subject Headings; WOS: Web of Science
